# Development of a Radiotherapy-Induced Wound Model in Wistar Rats: Simulating Post-Radiation Skin and Soft Tissue Complications for Therapeutic Evaluation

**DOI:** 10.3390/biomedicines14020415

**Published:** 2026-02-12

**Authors:** Stefana Avadanei-Luca, Bogdan Ionel Tamba, Irina Draga Caruntu, Simona Eliza Giusca, Andrei Daniel Timofte, Andrei Szilagyi, Ivona Costachescu, Maria Raluca Gogu, Andrei Nicolae Avadanei, Mihaela Pertea, Malek Benamor, Ionel Daniel Cojocaru, Mihai Liviu Ciofu, Viorel Scripcariu

**Affiliations:** 1 Department of Plastic Surgery, “Grigore T. Popa” University of Medicine and Pharmacy, 700115 Iasi, Romania; stefana_luca@umfiasi.ro (S.A.-L.); mihaela.pertea@umfiasi.ro (M.P.);; 2 Department of Pharmacology, “Grigore T. Popa” University of Medicine and Pharmacy, 700115 Iasi, Romania; 3 Center for Advanced Research and Development in Experimental Medicine (CEMEX), “Grigore T. Popa” University of Medicine and Pharmacy, 700115 Iasi, Romania; 4 Department of Morpho-Functional Sciences I, “Grigore T. Popa” University of Medicine and Pharmacy, 700115 Iasi, Romania; 5 Romanian Medical Science Academy, 030167 Bucharest, Romania; 6 Department of Vascular Surgery, “Grigore T. Popa” University of Medicine and Pharmacy, 700115 Iasi, Romania; 7 Elytis Radiotherapy and Oncology Center, 700714 Iasi, Romania; 8 Department of Oral and Maxillo-Facial Surgery, “Grigore T. Popa” University of Medicine and Pharmacy, 700115 Iasi, Romania; 9 Department of General Surgery, “Grigore T. Popa” University of Medicine and Pharmacy, 700115 Iasi, Romania

**Keywords:** radiotherapy, skin injury, soft tissue injury, post-radiation wound model, experimental wound

## Abstract

**Background/Objectives**: Radiotherapy can severely impair skin and soft tissue healing, particularly when high doses or subsequent surgical interventions are involved. Robust experimental platforms that replicate clinically relevant radiation-impaired wound healing remain limited. This study aims to establish a reproducible experimental model for radiation-induced cutaneous injury using contemporary clinical radiotherapy techniques. **Methods**: A Wistar rat model was developed using single-dose external beam irradiation delivered by clinical-grade volumetric modulated arc therapy (VMAT; 6 MV FFF), at doses of 20 Gy or 30 Gy. Animals were distributed in five distinct groups: G1—control, G2—20 Gy irradiation only, G3—20 Gy irradiation followed by excision, G4—excision only, G5—30 Gy irradiation only. Standardized full-thickness skin excision (1.5 × 1.5 cm) was performed one-week post-irradiation to simulate surgical intervention in pre-irradiated tissue. Animals were monitored for up to 42 days, through skin damage macroscopic scoring, body weight, hematological and biochemical parameters, and a qualitative histological exam. **Results**: Single-dose irradiation with 20 Gy induced moderate, self-limiting radiation dermatitis with complete healing. When combined with delayed excision, 20 Gy irradiation resulted in more severe and prolonged wound healing impairment, and transient systemic alterations. Excision alone produced controlled wounds with predictable healing. Exploratory observations following 30 Gy irradiation revealed severe cutaneous injury and marked systemic involvement, with a high mortality rate. **Conclusions**: This study establishes a foundational model for radiation-impaired wound healing using clinical-grade VMAT delivery and standardized delayed excision. The 20 Gy-based protocols provide an ethically sustainable and experimentally tractable platform for future mechanistic and therapeutic studies.

## 1. Introduction

Radiotherapy is a cornerstone in cancer treatment, effectively eliminating malignant tissues through high-energy ionizing radiation. However, this modality also damages adjacent healthy skin and subcutaneous tissues, often leading to acute and chronic complications that hinder wound healing [1,2]. Radiation-induced injuries may present as erythema, edema, necrosis, fibrosis, or chronic ulcers, significantly affecting tissue repair and patient’s quality of life [1].

At the cellular level, acute radiation injury manifests through characteristic morphological skin changes: epidermal thinning with basal cell depletion, increased nuclear–cytoplasmic ratio, and widened intercellular spaces [3], dermal fibroplasia with disorganized collagen deposition and microvessel damage leading to tissue hypoxia [4,5], and progressive loss of hair follicles and sebaceous glands compromising skin barrier function [6,7]. Inflammatory infiltrate follows a temporal pattern—initial neutrophil recruitment followed by mononuclear cell predominance during peak injury, with multiple inflammatory waves persisting for months in severe cases [8,9]. Chronic radiation damage, developing months to years post-treatment, is characterized by established fibrosis, telangiectasia, and in severe cases, necrosis with complete loss of epidermis and dermal appendages [10,11,12].

Experimental models of radiation-induced skin injury have been developed to study wound pathophysiology and evaluate therapeutic strategies, though significant methodological heterogeneity limits cross-study comparisons. A referential study established dose–response thresholds in rats using orthovoltage X-rays, demonstrating that single doses of 10–20 Gy produced reversible injury, whereas 30 Gy created ulcers persisting beyond six months—identifying the threshold for chronic non-healing wounds [13]. Rifkin et al. developed an athymic nude rat model using 30.4–76.5 Gy delivered via orthovoltage X-rays (250 kVp), recommending 41.5 Gy as optimal for reproducible ulceration; however, the immunodeficient phenotype alters inflammatory wound healing dynamics [14]. Jourdan et al. delivered 10–40 Gy soft X-rays followed by full-thickness wounding one-hour post-irradiation, demonstrating dose-dependent healing impairment through basement membrane disruption [15]. Similarly, Huang et al. used 50 Gy electron beam irradiation to create chronic radiation ulcers in Sprague-Dawley rats, establishing a model suitable for testing regenerative therapies [16]. More recently, Sheng et al. used high-dose-rate Ir-192 brachytherapy (90 Gy) to create chronic dermatitis progressing to fibrosis at 12 weeks [17], and Lawrence et al. validated a hairless mouse model receiving 30 Gy from 6 MeV electrons that closely mimics human radiation dermatitis grading [18].

Despite these advances, several limitations constrain the translational applicability of existing models. First, all published rat models employ orthovoltage X-ray units (100–320 kVp), gamma sources, or brachytherapy that diverge substantially from contemporary clinical megavoltage radiotherapy practice—orthovoltage X-rays exhibit 10–20% higher relative biological effectiveness than megavoltage photons, complicating dose translation [19]. Notably, no published rat model has employed volumetric modulated arc therapy (VMAT), the dominant technique in current clinical radiotherapy, representing a critical gap in the field. Second, few studies systematically assess systemic metabolic and hematological sequelae alongside local wound parameters, despite clinical recognition that radiation-impaired wounds often occur in patients with a compromised nutritional and metabolic status [20]. Third, the timing of surgical intervention relative to irradiation—a critical determinant of wound outcomes in patients undergoing post-radiation reconstruction—remains inconsistently standardized across published models, with intervals ranging from immediate post-irradiation wounding to a several weeks delay.

Within this context, the aim of the present study was to develop a new standardized protocol for radiation-induced wound injury in an experimental model, providing a better simulation of this particular type of lesion with potential applicability in purposeful research evaluating biomaterials or testing specific therapeutics for healing.

## 2. Materials and Methods

### 2.1. Animal Model

The study was conducted on 28-week-old adult male Wistar rats (340–450 g), housed in individually ventilated cages (IVCs) within the animal facility at the Advanced Research and Development Center for Experimental Medicine “Prof. Ostin C. Mungiu”—CEMEX. They were maintained under standard husbandry conditions, including controlled room temperature (20 ± 4 °C), relative humidity (50 ± 5%), and a controlled light–dark cycle with unrestricted access to water and standard laboratory chow. All experimental protocols complied with Directive 2010/63/EU, ARRIVE Guidelines, the European Convention for the Protection of Vertebrate Animals (Council of Europe No. 123/1985), and national regulations. Approval was obtained from the Ethics Committee of “Grigore T. Popa” University of Medicine and Pharmacy, Iași (no. 253/11 January 2023), and the Romanian National Sanitary Veterinary and Food Safety Authority (no. 30/28 March 2023).

The experimental design included five distinct groups, each group comprising 8 animals: G1—control (no irradiation or excision), G2—20 Gy irradiation only, G3—20 Gy irradiation followed by excision, G4—excision only, G5—30 Gy irradiation only. Animals were randomly allocated to experimental groups prior to study initiation. To minimize observer bias, all wound assessments were performed independently by two members of the team, with discrepancies resolved by consensus.

Irradiation was performed using a linear accelerator, targeting a 1.5 × 1.5 cm area on the dorsal paravertebral skin (T10–L4). A single dose was delivered: 20 Gy for G2 and G3, and 30 Gy for G5 to induce more severe injury. G3 and G4 also underwent surgical excision (15 mm diameter, 0.1 mm thickness) to create full-thickness wounds. According to experimental protocol, the monitoring timeline was 42 days. Blood and skin samples were collected for all animals in G1, G2 and G4 at the end of the timeline, following their sacrifice. For G3, taking into consideration the significantly impaired wound evolution, the experimental protocol was adapted as follows: the animals were sacrificed in two subsets: 4/8 animals on day 28 (with histopathological assessment only) and 4/8 animals on day 42 (with both blood and histopathological assessment). Particularly, due to the high mortality in Group 5 (7/8 animals, 87.5%), with only one animal surviving beyond day 42 and being monitored for 150 days to characterize the chronic wound phenotype, Group 5 was excluded from all comparative statistical analyses, being considered as an exploratory pilot group.

### 2.2. Irradiation Protocol

**Figure 1:**
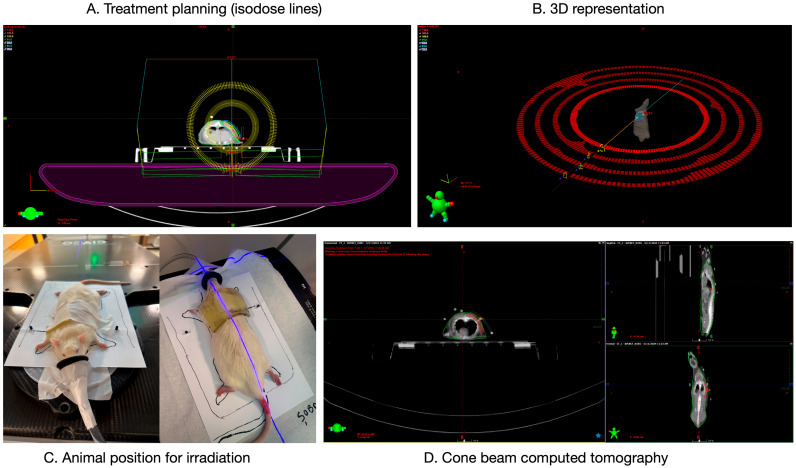
Irradiation protocol: (**A**) planning target volume in Eclipse software (version 18.0); (**B**) 3D representation; (**C**) radiotherapy positioning of the animal; (**D**) cone-beam computer tomography images obtained from different sections of the target volume, displayed in transverse, sagittal, and frontal planes.

Irradiation procedures were performed at the Elytis Radiotherapy Medical Center using a Halcyon linear accelerator (Varian Medical Systems Inc., Palo Alto, CA, USA; 6 MV FFF, 800 MU/min). Anesthesia was induced with 5% isoflurane and maintained at 2% via inhalation mask, with animals in prone position. All subjects underwent computer tomograph (CT) simulation (Somatom go.Sim, Siemens, Erlangen, Germany) with 3 mm slice thickness. Planning target volume (PTV) delineation and treatment planning were performed using Eclipse software (version 18.0, Varian Medical Systems Inc., Palo Alto, CA, USA). A single treatment plan per dose group (20 Gy or 30 Gy) was optimized and approved by both the radiation oncologist and physicist. Dose delivery employed VMAT with 4 arcs for 20 Gy and 6 arcs for 30 Gy (Figure 1). Animals were positioned identically to the simulation. A kilovoltage cone-beam computed tomography (kVCBCT) scan was performed prior to treatment to verify alignment. Irradiation lasted approximately 10 min per animal, with total session time (including preparation) of 30–45 min per animal.

### 2.3. Wound Induction

To enhance simulation of post-radiation subcutaneous tissue damage, a standardized 1.5 × 1.5 cm full-thickness skin excision was created in a subset of groups one week after irradiation, as the 20 Gy dose alone did not induce deep ulceration. A patented wound-covering device (RO 131700 B1) was subsequently applied to protect the defect and ensure standardized monitoring of healing. Anesthesia was induced with 5% isoflurane and maintained at 2%, with adequacy confirmed by loss of pedal reflexes. Following excision, hemostasis was achieved as required, and six transcutaneous fixation points were created using a 16G needle to introduce piercings for device stabilization. Each piercing was secured within the device grooves and capped with metallic fasteners to maintain dressing stability. Postoperatively, analgesia was administered via intraperitoneal Tramadol (12.5 mg/kg), and animals were individually housed and monitored until full recovery from anesthesia.

### 2.4. Assessment Protocols

Standardizing the assessment of radiation effects and the ensuing wound development was the aim of the evaluation techniques. Wound progression and systemic effects were monitored through macroscopic lesion assessment, blood analysis, and a qualitative histological exam. General health, including weight and behavior, was assessed regularly. Wounds were inspected daily for one week, then weekly. The macroscopic changes in the wound aspect were evaluated using a validated semi-quantitative scoring scale ranging from 1.0 (no effect) to 5.5 (necrosis) [20]. This system captured the progressive spectrum of radiation-induced skin damage, including erythema, desquamation, crusting, scabbing, ulceration, and ultimately full-thickness loss or necrosis [20]. In addition to the semi-quantitative scoring, maximum wound diameter was recorded manually at each assessment timepoint. Manual measurements were performed using a ruler, independently by two members of the research team (S. A.-L., A. S.). If differences greater than 2 mm were recorded, the consensus was obtained by the two evaluators working together, thus eliminating human errors due to the inherent subjectivity in manual measurements. However, data was not subjected to formal statistical analysis due to variability in wound geometry and contraction patterns inherent to rodent skin healing.

Terminal cardiac puncture under isoflurane anesthesia was performed to collect blood for subsequent biochemical and hematological analyses. Blood samples were collected into 3 mL clot activator tubes for biochemical analyses and 2 mL K3-EDTA tubes for hematological evaluation. To obtain the serum for biochemical analyses, the collected blood samples were allowed to clot at room temperature for 30 min and were subsequently centrifuged at 1500× *g* for 15 min at 4 °C. Serum biochemical parameters, including alanine aminotransferase (ALT/ALAT), aspartate aminotransferase (AST/ASAT), creatinine, total cholesterol, glucose, albumin, total protein, urea, and bile acids, were analyzed using Cormay Accent 200 automated biochemical analyzer (PZ Cormay S.A., Lomianki, Poland). All measurements were performed using commercially available Cormay reagents and assay kits specific to each parameter, in accordance with the manufacturer’s instructions. Hematological analyses evaluated red blood cell count (RBC), hemoglobin, hematocrit, platelet count, white blood cell (WBC) and differential leukocyte counts (neutrophils and lymphocytes). Analyses were conducted using IDEXX ProCyte One automated hematology analyzer (IDEXX Laboratories, Inc., Westbrook, ME, USA), with IDEXX reagents and assay kits, in accordance with the manufacturer’s instructions. Reference ranges for hematological and biochemical parameters were obtained from published normative data for Crl:WI (Han) Wistar rats [21]. Values from the control group (G1) were compared against these established reference intervals to confirm consistency with published physiological ranges.

Animals were euthanized via isoflurane overdose.

Tissue samples obtained by excision of full-thickness skin biopsies with a 0.5–1 cm margin were fixed in 10% buffered formalin, paraffin-embedded, and stained with hematoxylin and eosin (H&E) and trichrome stain for the qualitative histological evaluation. The assumed qualitative histological assessment of the induced lesions and their healing progression was intended only to support the macroscopic and clinical evaluation of tissue damage and repair, without performing measurements of epidermal or dermal thickness, collagen density, fibrosis extent, or vascularization.

### 2.5. Statistical Analysis

Statistical analyses were performed using Python 3.12 with SciPy (v1.13) and Pingouin (v0.5) packages. Data are expressed as mean ± standard deviation (SD). Normality was assessed using the Shapiro–Wilk test, and homogeneity of variances was evaluated using Levene’s test.

For body weight analysis with repeated measurements, a two-way mixed-model ANOVA was performed with groups (G1–G4) as the between-subjects factor and time as the within-subjects factor. Greenhouse–Geisser correction was applied when sphericity was violated (Mauchly’s test *p* < 0.05). One-way ANOVA with Tukey’s HSD post hoc test was performed at each individual timepoint. For blood chemistry parameters, one-way ANOVA with Tukey’s HSD post hoc test was used for normally distributed data with homogeneous variances, or the Kruskal–Wallis test with Dunn’s post hoc test (Bonferroni correction) when assumptions were violated. Skin damage macroscopic scores were analyzed using the Kruskal–Wallis H test due to their ordinal nature, with Mann–Whitney U test and Bonferroni correction for post hoc pairwise comparisons. Statistical significance was set at α = 0.05 (two-tailed). Effect sizes are reported as partial eta-squared (η^2^p) for ANOVA.

Group 5 (30 Gy; *n* = 1 survivor) was excluded from comparative analyses and is presented descriptively.

## 3. Results

### 3.1. Irradiation

The 30 Gy dose markedly impaired animal quality of life, leading to weight loss, anorexia, and general debilitation. Due to these severe effects, G5 was sacrificed early, with only one animal monitored for five months to assess chronic radiation-induced changes. In contrast, exposure to 20 Gy dose did not result in a decisive impairment of systemic status and quality of life, preserving the wound-healing capacity of irradiation-induced lesions, irrespective of excision.

### 3.2. Wound Healing Rates

#### 3.2.1. Weight Status

**Figure 2:**
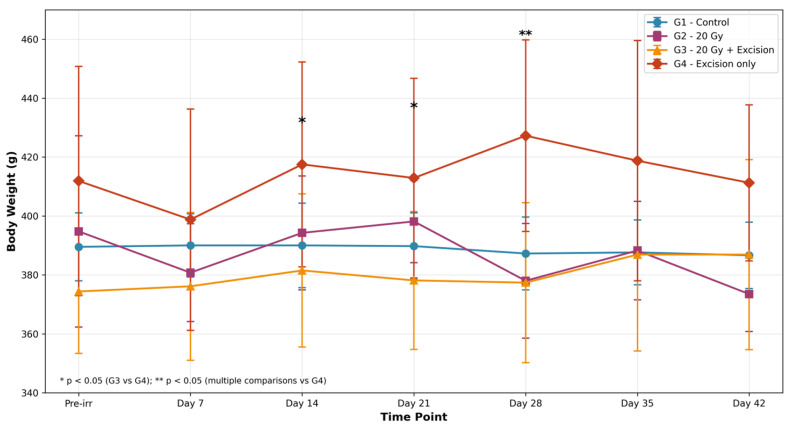
Body weight changes across experimental groups. Data are presented as mean ± SD.

G1 maintained stable weight throughout the study. G2 exhibited minor fluctuations without significant deviations. G3 showed some variability, suggesting inconsistent recovery likely due to surgical intervention. In contrast, G4 demonstrated a more stable weight trajectory, with fewer fluctuations than G2 (Figure 2, Table 1).

In G5, seven of eight animals (87.5%) reached endpoints (over 20% body weight loss and persistent anorexia for more than 72 h) between days 14 and 35, thus leading to marked behavioral deterioration (hunched posture, reduced mobility, piloerection). However, the single surviving animal presented minimal changes in the weight status (Table 1).

Mixed ANOVA revealed a significant time effect (F = 3.255, *p* = 0.037, Greenhouse–Geisser corrected; η^2^p = 0.104), but no significant group effect (F = 2.926, *p* = 0.051) or group × time interaction (F = 0.958, *p* = 0.480). One-way ANOVA at individual timepoints showed significant differences at day 14 (F = 3.076, *p* = 0.044), day 21 (F = 3.392, *p* = 0.032), and day 28 (F = 4.708, *p* = 0.012), with post hoc analysis indicating G4 was significantly heavier than G3 at these timepoints (*p* < 0.05).

#### 3.2.2. Macroscopic Findings

**Figure 3:**
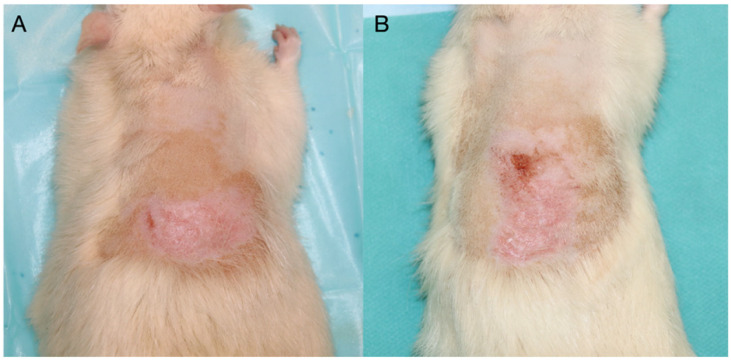
Group 2—(**A**). Day 14, (**B**). Day 28.

In the absence of any intervention, G1 showed normal skin without any spontaneous lesions developed over the experiment. In G2, no noticeable changes were observed during the first week. Between days 7–28, five animals developed mild erythema and skin dryness, (score 1.5). while three showed moderate erythema, dryness, and superficial scabbing from day 14 (score 2.0) (Table 2). Dermatitis regressed after day 28, with complete macroscopic healing by day 42 in all cases (Figure 3).

**Figure 4:**
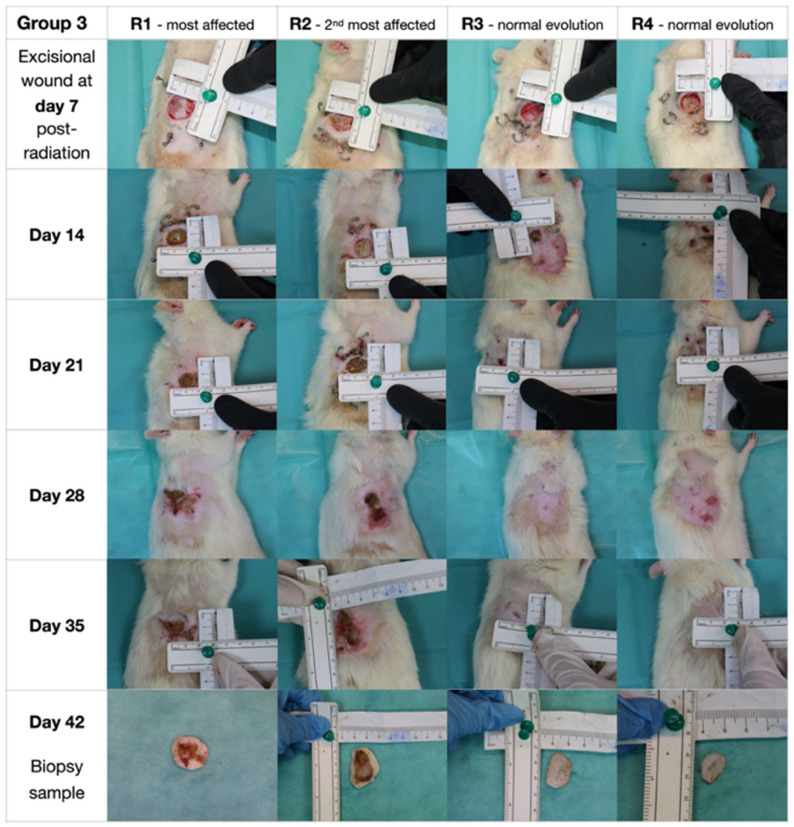
Group 3—20 Gy irradiation + excision. R1 and R2—most affected animals; R3 and R4—normal evolution of the lot.

G3 presented similar results to G2 after irradiation; thus, a deliberate full-thickness cutaneous defect was created to replicate severe tissue injury. Two animals developed deeper combined post-irradiation and post-excision lesions. Early findings (day 7) showed minimal erythema and dryness (score 1.5), progressing to moist desquamation and ulceration by day 14, and extensive scabbing by day 21 (score 4.5). Necrosis with central sloughing and partial contraction was observed at day 28, while day 35 revealed extensive tissue loss with gradual reduction in wound size (score 5.0). By day 42, we noted peripheral epithelialization, reflecting chronic injury and delayed remodeling. In contrast, other animals exhibited faster recovery, with progressive contraction, re-epithelialization, and near-complete closure by day 35, and visual assessment at day 42 showed smaller, well-healed scars (Figure 4).

**Figure 5:**
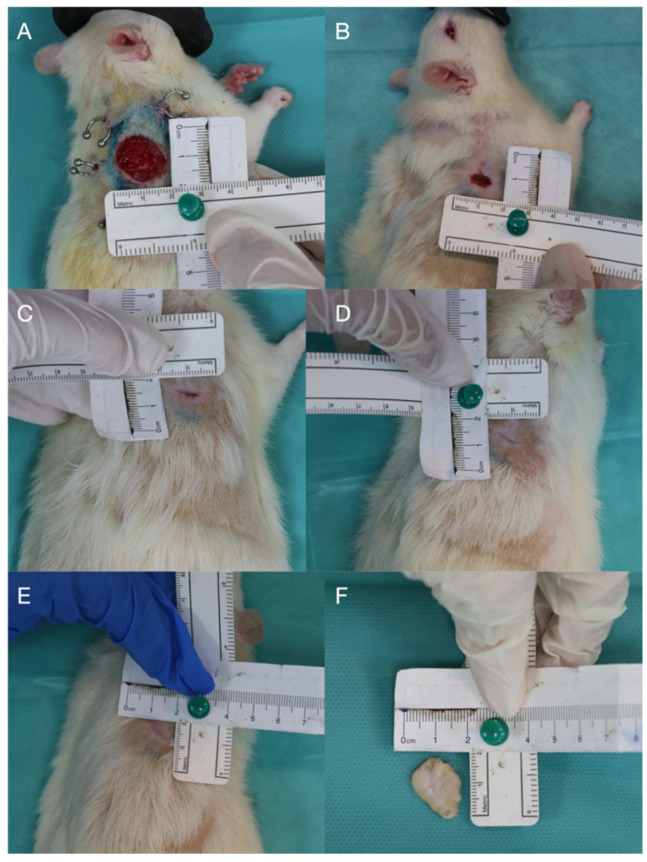
Group 4: non-irradiated, with excision; (**A**) day 7, post-excision; (**B**) day 14; (**C**) day 21; (**D**) day 28; (**E**) day 35; (**F**) day 42—biopsy sample.

G4 demonstrated gradual healing of a controlled full-thickness skin defect. Initial injury (score 5.0) progressed to central necrosis by day 7 (score 5.5), followed by partial epithelialization and reduced inflammation by day 14 (score 2.0). Near-complete closure still presenting a central ulceration surrounded by residual scarring was observed on day 28. The ulceration progressively diminished, on day 42 only a minor central defect being observed (Figure 5).

**Figure 6:**
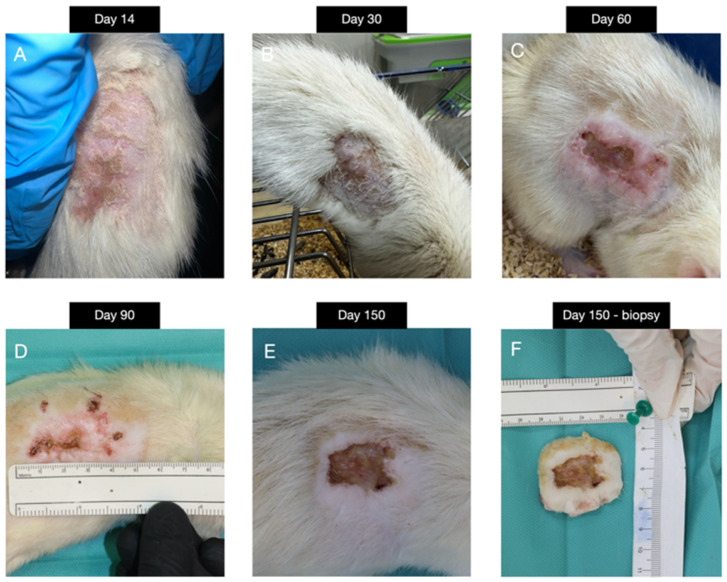
Group 5—irradiated with 30 Gy; (**A**) day 14 post-irradiation; (**B**) day 30; (**C**) day 60; (**D**) day 90; (**E**) day 150; (**F**) day 150—biopsy sample.

The following findings from G5 (30 Gy) obtained from a single surviving animal are presented as preliminary exploratory observations of chronic radiation injury phenotype. G5 illustrated the severe, protracted course of radiation-induced dermatitis, monitored over five months. Initial changes included moderate erythema and dryness (score 2.0), progressing by day 30 to dry desquamation with crusting and fibrotic margins (score 3.0). By day 60, lesions showed moist desquamation, deep scabbing, and ulceration with hyperpigmented, thickened edges (score 4.5). At day 90, the wound displayed central crusting with peripheral vascularized granulation tissue, while by day 150 only partial healing was achieved, with persistent necrosis, tissue loss, and incomplete epithelialization (score 5.0) (Figure 6).

**Figure 7:**
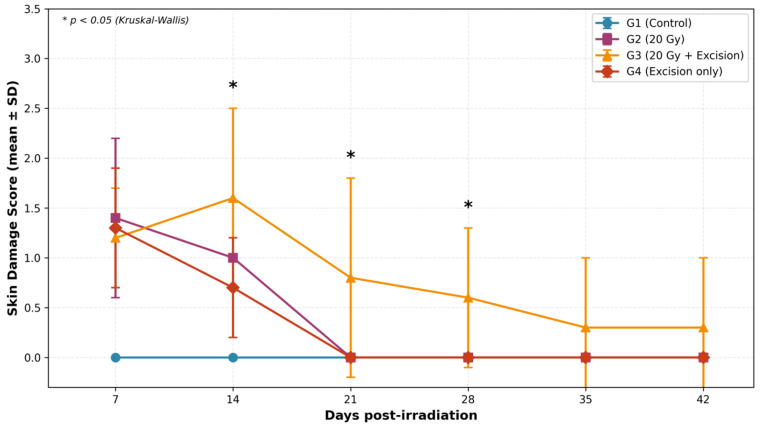
Dynamics of skin damage scores across experimental groups.

Statistical analysis showed that skin damage scores differed significantly among groups at days 14, 21, and 28 (Kruskal–Wallis: *p* < 0.05 for all) (Figure 7, Table 2). At days 7 and 14, all intervention groups (G2, G3, G4) showed significantly higher scores than controls (*p* < 0.05). By day 21, both G2 and G4 achieved complete resolution (*p* < 0.05 vs. G1 and G3). G3 showed the most prolonged clinical response, with residual macroscopic alterations persisting through day 42 (0.3 ± 0.7), though not significantly different from other groups (*p* = 0.495).

### 3.3. Biochemical and Hematological Parameters

Liver enzymes (ASAT/TGO and ALAT/TGP) in G2 showed slight increases compared to other groups (*p* = 0.002, *p* < 0.001, respectively), while G3 exhibited more marked abnormalities, including low albumin (*p* < 0.001 vs. G1), reduced glucose levels (90.1 ± 26.8 mg/dL), and elevated bile acids (63.6 ± 15.4 µmol/L, *p* < 0.001). G4 presented milder changes—mainly low albumin and occasional enzyme elevation—suggesting a systemic effect of excision alone (Table 3).

Regarding hematological parameters, G3 exhibited signs of mild anemia with reduced RBC counts (*p* < 0.001), decreased hemoglobin (*p* < 0.001), and borderline low haematocrit (*p* = 0.003) compared to controls. Platelet counts showed a trend toward thrombocytosis in G3, though this did not reach statistical significance compared to G1 (*p* = 0.126). WBC and differential leukocyte population (neutrophils, lymphocytes) counts remained within normal ranges across G1–G4 (*p* > 0.05), suggesting that the systemic inflammatory response did not manifest as peripheral leukocytosis. G2 and G4 showed minimal hematological deviations from controls, indicating that the combined radiation–surgical injury in G3 produced a synergistic effect on haematopoiesis.

G5 demonstrated the most severe alterations, including elevated liver enzymes, thrombocytosis, and anemia, reflecting the systemic impact of high-dose irradiation.

### 3.4. Histological Changes

**Figure 8:**
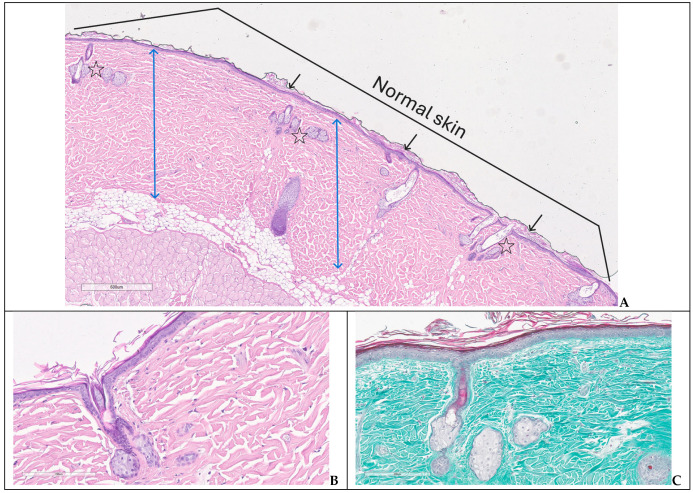
Normal structure of the skin—group 1: (**A**) keratinized epidermis (black arrows), dermis (two-headed blue arrows) and skin appenddages—hair follicles and sebaceous glands (stars); (**B**,**C**) detailed image of normal skin and appenddages (H&E, trichrome).

In G1, the microscopic assessment performed at day 42 showed normal structure of epidermis and dermis, preserved hair follicles, sebaceous and sweat glands (Figure 8A–C).

**Figure 9:**
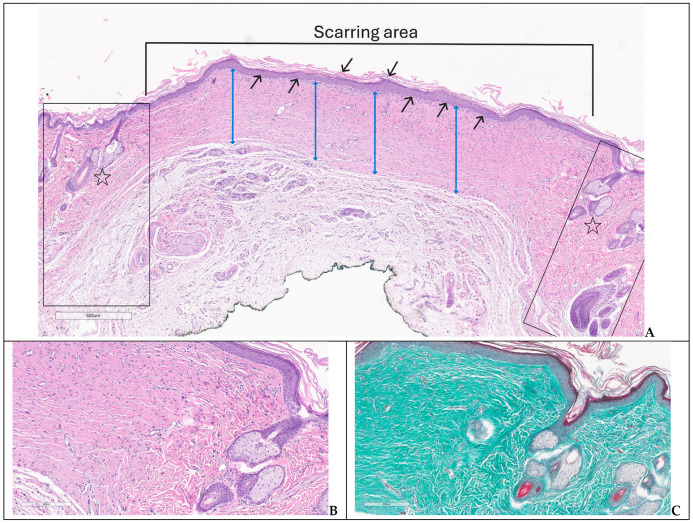
Immature scar with complete re-epithelialization—group 2: (**A**) regenerated epidermis with keratin (black arrows) and hypercellular, highly collagenized dermis (two-headed blue arrows) borded by normal skin (boxes) with appendages (stars) (H&E); (**B**,**C**) detailed images of the scarring area and normal skin with preserved skin appendages at the wound borders (H&E, trichrome).

In G2, also sacrificed at day 42, the assessment of the skin defect showed an immature scar covered by completely regenerated epidermis with normal structure and lamellar keratin on the surface, and an intensely collagenized dermis with thick collagen bundles oriented parallel with the epidermis, hypercellular due to the increased number of fibroblasts (some reactive), fibrocytes and lymphocytes. Skin appendages were present only in the normal skin preserved at the wound borders (Figure 9A–C).

**Figure 10:**
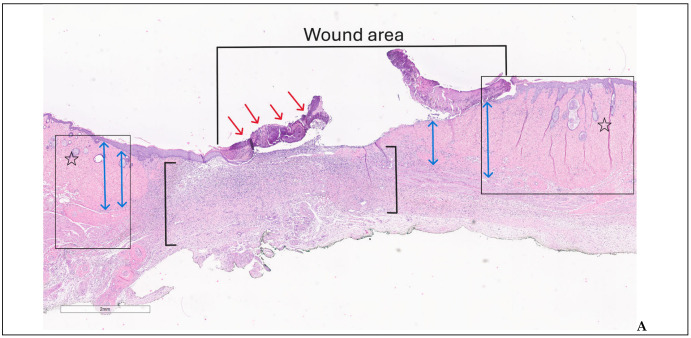

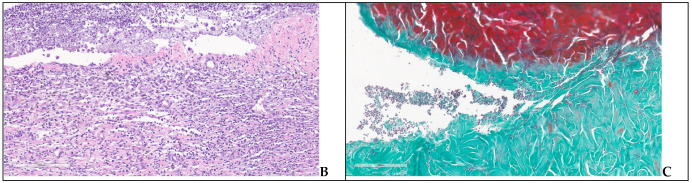
Wound area—group 3, day 28: (**A**) central ulceration covered by crust (red arrows), abundant inflammatory infiltrate throughout dermis and hipodermis (black square brackets), adjacent normal skin (boxes) with collagenized dermis (two-headed blue arrows) and appendages (stars) (H&E); (**B**,**C**) detailed images illustrating the dermal polymorphic inflammatory infiltrate ((**B**)—H&E) and partially detached crust ((**C**)—trichrome).

**Figure 11:**
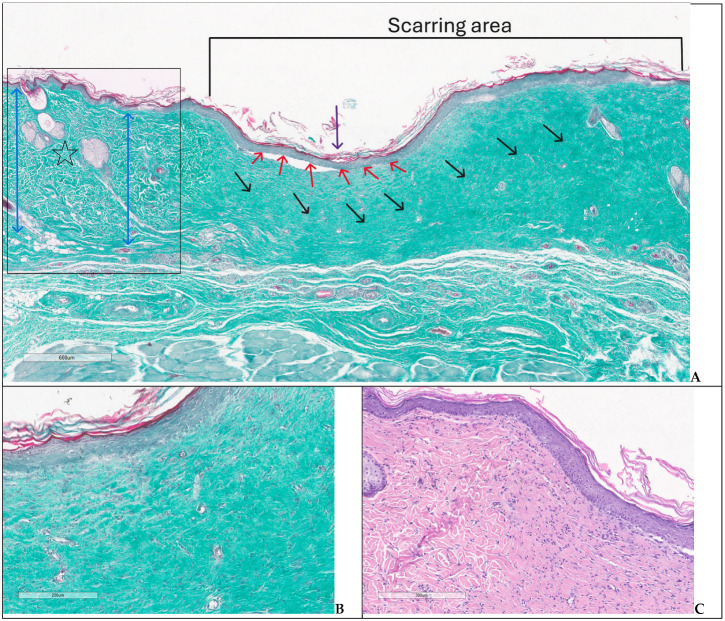
Scarring area—group 3, day 42: (**A**) re-epithelialization maintaining a central depressed area (dark purple arrow pointing down), thick epidermis (red arrows), dermis with compacted collagen bundles (obliques black arrows), on the left side transition between scarring area and normal skin (box) with different collagen bundle distribution (two-headed blue arrows) and appendages (stars) (trichrome); (**B**,**C**) detailed images showing regenerated epidermis and underlying dermis with numerous fibroblasts and capillaries (trichrome, H&E).

In G3 on day 28, two of the experimental animals showed an extensive ulceration covered by thick crust, underlying abundant polymorphic inflammatory infiltrate with numerous intact and altered PMNs, edema, and neoangiogenesis—specific aspects for an immature granulation tissue (Figure 10A–C). The inflammatory infiltrate was observed deeper in the dermis and hypodermis, separating the muscle fibers at the base of the skin defect; the arterial vessels in the hypodermis showed recent thrombosis and vasculitis. On day 42, the animals had partial healing, consisting of epithelial regeneration initiated from the wound margins towards the central area that appeared sunken, recessed, like a small pit; beyond the dermal epidermal junction, the dermis showed thin, compacted collagen bundles, with numerous reactive fibroblasts, moderate chronic inflammatory infiltrates, and newly formed capillaries; regenerated hair follicles and sebaceous glands were noted only at the wound boundaries (Figure 11A–C).

**Figure 12:**
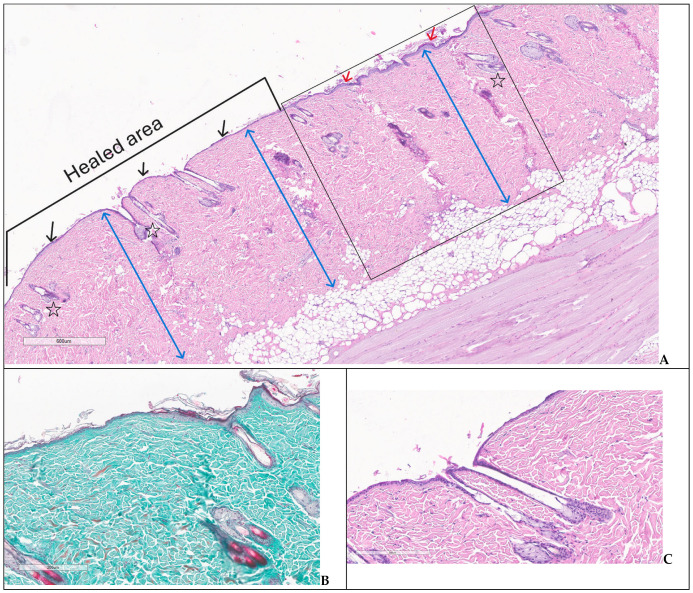
Complete healing—group 4: (**A**) restored integrity of the previous wound area with thin keratinized epidermis (black arrows), normal dermis (two-headed blue arrows) and newly formed skin appendages—hair follicles and sebaceous glands (stars) bordered by normal skin (box)—note the slightly thicker epidermis (red arrows) and similar aspect of the dermis (two-headed blue arrows) (H&E); (**B**,**C**) detailed images for healing process with complete structural restoration of skin (trichrome, H&E).

Group 4 presented complete re-epithelization, normal superficial and deeper dermis including mature skin appendages, hypodermis with white adipocytes arranged in compact lobules, focally with a tendency to ascend into the dermis and striated muscle tissue arranged in bundles (Figure 12A–C).

**Figure 13:**
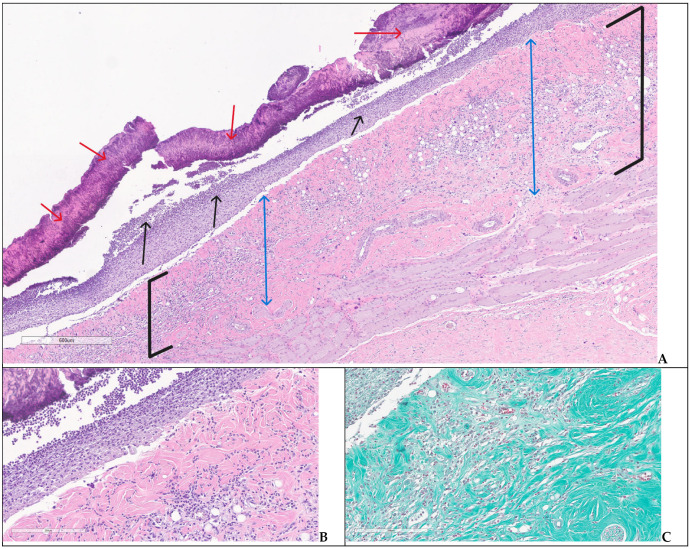
Severe skin damage—group 5: (**A**) ulceration covered by thick crust (red arrows) with subjacent acute inflammatory infiltrate (black arrows), heavy polymorphic inflammatory infiltrate in dermis (black square brackets, two-headed blue arrows) (H&E); (**B**,**C**) detailed images for the crust and dermal inflammation, and newly formed capillaries (H&E, trichrome).

In Group 5, the histological evaluation performed on day 150 indicated: peripheral re-epithelialization of the defect, maintaining a central ulceration covered by a fibrinous-leukocytic crust and a thick band of acute inflammatory infiltrate; young granulation tissue at the base of the lesion, with numerous capillaries, polymorphic inflammatory infiltrate; intensely collagenized dermis composed by thick bundles separated by polymorphic inflammatory infiltrate; no hair follicles and sebaceous glands; bundles of striated muscle surrounded by fibrosis (Figure 13A–C).

## 4. Discussion

This study establishes a Wistar rat model of radiation-induced cutaneous injury that addresses several methodological gaps in the existing literature. The principal innovation is the use of clinical-grade VMAT with image-guided verification—a technique that, to the best of our knowledge, has not been previously applied to rodent skin injury modeling despite representing the dominant modality in contemporary clinical radiotherapy.

The radiation delivery method and timing fundamentally distinguish our approach from established models. One of the classical models [13] used orthovoltage X-rays to establish dose–response thresholds (10–30 Gy) in rats, identifying 30 Gy as the threshold for chronic ulceration persisting beyond six months. Consistent with these findings, our 30 Gy protocol produced chronic non-healing wounds in the single surviving animal (day 150 skin damage score: 3.0), while our 20 Gy protocol produced reversible injury with complete resolution by day 42 (skin damage score: 0). Another study [14] employed an XRAD-320 image-guided stereotactic irradiator operating at 250 kVp in athymic nude rats, recommending 41.5 Gy for optimal ulcer formation with minimal wound contraction. Notably, our combined 20 Gy + excision approach achieved comparable wound severity (with a peak in skin damage score of 4.5–5.0 at days 21–35 in the most affected animals) at approximately half the radiation dose, suggesting that the delayed excision amplifies radiation injury without requiring prohibitively high doses. Jourdan et al. [15] delivered 10–40 Gy using soft X-rays in a combined radiation-wound model with wounds created one-hour post-irradiation. In contrast, our one-week delayed excision produced more severe combined injuries at lower radiation doses (20 Gy vs. their 40 Gy threshold for significant impairment). Huang et al. [16] utilized 50 Gy electron beam irradiation (6 MeV) to create chronic radiation wounds requiring 8–12 weeks for ulcer formation. Our 20 Gy + excision protocol achieved ulceration within 2–3 weeks, providing a more rapid experimental timeline. Sheng et al. [17] employed high-dose-rate Ir-192 brachytherapy at 90 Gy to induce dermatitis progressing to fibrosis over 12 weeks. It is worth mentioning that we obtained fibrosis in immature scars confirmed histologically, in a shorter time, by using a low irradiation dose (20 Gy) alone or combined with excision. A recent paper [18] validated a 30 Gy mouse model using 6 MeV electrons that closely mimicked human radiation dermatitis grading—the only prior model using megavoltage electrons—but their study did not include systemic metabolic assessment. All these models rely on orthovoltage X-rays (100–320 kVp), electron beams, or brachytherapy sources that exhibit fundamentally different dose deposition characteristics compared to the up-to-date methods applied in human radiotherapy treatment.

The translational significance of this distinction warrants emphasis. Orthovoltage X-rays demonstrate 10–20% higher relative biological effectiveness (RBE) than megavoltage photons due to increased photoelectric absorption and higher linear energy transfer [19]. This necessitates RBE adjustment when extrapolating experimental doses to clinical scenarios, introducing uncertainty into a perfect therapeutic conversion from animals to humans. It is important to highlight that orthovoltage sources produce maximum dose at the skin surface with rapid falloff, whereas megavoltage beams exhibit skin sparing with dose buildup—a characteristic that fundamentally alters the depth–dose relationship. By employing VMAT with 6 MV flattening-filter-free (FFF) photons and kV-CBCT verification, our model replicates the dose distribution patterns that patients receiving modern conformal radiotherapy actually experience, potentially improving the predictive validity for clinical radiodermatitis.

Nonetheless, the deliberate selection of single-dose irradiation in our model should be discussed. While clinical radiotherapy typically employs fractionated regimens (e.g., 50–70 Gy in 1.8–2.0 Gy fractions), single-dose protocols offer several experimental advantages for model design, as follows: (1) precise dose–response characterization without confounding from inter-fraction repair; (2) reduced inter-animal variability arising from multiple anesthesia exposures; (3) logistical feasibility and reduced animal handling stress; and (4) established precedent in experimental radiobiology [13,14,15,16,17,18].

Although most of the already proposed experimental models used radiation alone without surgical wounding [14,16,17], the timing of surgical intervention relative to irradiation critically determines wound outcomes.

In our study, statistical analysis confirmed that this timing amplifies injury severity. G3 demonstrated significantly higher skin damage scores compared to G4, with elevated values at days 14, 21, and 28 (*p* < 0.05). Furthermore, G3 animals showed significantly lower body weights than G4 at days 14, 21, and 28 (*p* < 0.05), indicating that pre-irradiation impairs not only local wound healing but also systemic recovery from surgical trauma. This timing allows the 20 Gy dose—which alone produces only moderate, reversible dermatitis—to manifest as severe, delayed healing wounds when combined with surgical trauma, mimicking the surgical wound complications observed in patients undergoing post-radiation reconstruction [20].

A distinctive feature of our model is the systematic assessment of metabolic and hematological parameters alongside local wound evaluation. Clinical radiation injury frequently occurs in patients with compromised nutritional status, where systemic metabolic imbalance—hypoalbuminemia, glucose dysregulation, anemia—contributes to impaired wound healing [20]. Previous rat models have primarily focused on local wound parameters (wound area, histopathology, tensile strength) without pointing to this systemic dimension. The significant hypoalbuminemia, reduced glucose levels, and elevated liver enzymes observed in G3 parallel the metabolic profiles seen in clinical radiation injury patients and enable evaluation of therapeutic interventions targeting both local and systemic pathology. Specifically, compared to the other groups (including control), G3 exhibited significant hypoalbuminemia (*p* < 0.001), reduced glucose levels (*p* < 0.001), elevated bile acids (*p* < 0.001), and elevated liver enzymes (*p* < 0.01). Notably, excision alone that characterized G4 did not produce significant hypoalbuminemia (*p* = NS vs. G1), confirming that the systemic metabolic disturbance in G3 reflects a synergistic effect of combined radiation and surgical injury rather than surgical stress alone. Hematological evaluation showed additional evidence of systemic compromise in the combined injury. Statistical intergroup analysis also showed that G3 exhibited signs of mild anemia with significantly reduced RBC counts (*p* < 0.001), decreased hemoglobin (*p* < 0.001), and borderline low hematocrit (*p* = 0.003), while platelet counts showed a trend toward thrombocytosis (*p* = 0.126). Importantly, WBC and differential leukocyte count remained within normal ranges (*p* > 0.05), indicating that the systemic stress response in G3 did not manifest as peripheral leukocytosis but rather as a chronic consumptive process affecting erythropoiesis. The absence of significant hematological alterations in G2 (similar to another study [14]) and G4 further supports the complementary pathophysiology of combined radiation–surgical injury.

The within-group heterogeneity observed in G3, where some animals developed severe non-healing wounds while others achieved near-complete closure by day 35, merits specific comment. Similar heterogeneity has been already reported in the mainstream [14,15]. Rather than representing a model limitation, this variability mirrors the clinical heterogeneity observed after surgery in previously irradiated fields, where patient outcomes range from uncomplicated healing to chronic wound breakdown despite similar treatment parameters [22]. Contributing factors likely include individual differences in local tissue perfusion, magnitude of inflammatory response, subclinical bacterial colonization, and microenvironmental variations within the irradiated field.

Despite the concept, our research has some limitations we acknowledge.

First, wound assessment relied on a validated semi-quantitative scoring system for skin damage, intentionally applied because it includes multiple clinically relevant parameters. We did not use digital planimetry or automated systems because edema, degree of tissue congestion, and other signs of skin injury should be assessed in real time during the experiment.

Second, the high mortality in G5 limited observations to a single surviving animal, precluding statistical analysis of chronic wound phenotypes. Consequently, we chose to present these observations as exploratory data, illustrative of severe radiation injury, without an evidence-based value for a definitive model characterization. However, protocol modifications—including dose fractionation, enhanced supportive care, or reduced radiation fields—would be required before the 30 Gy dose level can be recommended for routine use.

Third, inherent anatomical differences between rodent and human skin constrain direct clinical translation. Rat skin is thinner, more loosely attached, and heals predominantly by wound contraction facilitated by the panniculus carnosus muscle—mechanisms that differ from human re-epithelialization-dominant healing [23]. These species-specific differences affect wound closure kinetics and must be considered when extrapolating therapeutic efficacy to clinical settings.

Fourth, although macroscopic and histological assessments were performed, we did not include quantitative histomorphometric measurements of key skin parameters (epidermal thickness, collagen density, vascular density) or a quantitative assessment of immunohistochemical markers to characterize the tissular and molecular balance between injury and healing. We included the presentation of histological changes to illustrate the timeline of the healing process after applying three different models of injury, according to the literature focusing on repair sequence. As the next step in developing our experimental model, we intend to incorporate quantitative analysis of fibrosis, inflammation, and angiogenesis, supported by distinctive immunohistochemical biomarkers for these processes (collagen IV and metalloproteinases for fibrosis, CD68 for macrophages in the inflammatory infiltrate and surrounding healing areas, CD31 and α-SMA for newly formed vessels). This approach may contribute to a deeper understanding of the molecular pathways underlying radiation-impaired wound healing.

## 5. Conclusions

In summary, our model offers several methodological advantages compared to existing approaches: (1) clinical-grade VMAT delivery with kV-CBCT verification ensures dosimetric precision and clinical relevance unmatched by orthovoltage- or brachytherapy-based models; (2) the standardized delayed excision paradigm creates clinically relevant combined injuries modeling post-radiation surgical scenarios, with statistically demonstrated amplification of injury severity; (3) integrated systemic metabolic assessment enables evaluation of the interconnected local–systemic pathophysiology. These features position the model as a translational bridge between basic radiobiology studies and potential clinical therapeutic development.

The 20 Gy protocol, alone or combined with full-thickness excision, provides a standardized and ethically sustainable model for studying radiation-impaired wound healing, with the caveat that quantitative wound measurements and mechanistic analyses should be incorporated to maximize translational value.

## Figures and Tables

**Table 1:** Animal welfare mirrored by weight variations.

Groups	Weight (Mean ± SD)						
	Pre-Irradiation	Day 7	Day 14	Day 21	Day 28	Day 35	Day 42
G1	389.5 ± 11.5 g	390.0 ± 10.7 g	390.0 ± 14.4 g	389.8 ± 11.3 g	387.2 ± 12.3 g	387.6 ± 11.0 g	386.6 ± 11.3 g
G2	405.5 ± 19.2 g	375.8 ± 20.7 g	385.2 ± 15.3 g	370.0 ± 23.2 g	352.8 ± 19.0 g	360.0 ± 0.0 g	358.0 ± 18.0 g
G3	374.4 ± 19.6 g	376.1 ± 23.5 g	381.5 ± 24.3 g	378.1 ± 21.9 g	377.4 ± 25.4 g	386.9 ± 30.5 g	386.9 ± 30.6 g
G4	411.9 ± 36.4 g	398.8 ± 35.1 g	417.5 ± 32.6 g	412.9 ± 31.7 g	427.2 ± 28.2 g	418.8 ± 35.3 g	411.3 ± 23.0 g
*p*-value	0.085	0.284	0.044 *	0.032 *	0.012 *	0.235	0.167
	**Pre-Irradiation**	**Day 30**	**Day 60**	**Day 90**	**Day 110**	**Day 130**	**Day 150**
G5^#^	445.0 ± 0.0 g	432.0 ± 0.0 g	412.0 ± 0.0 g	411.0 ± 0.0 g	420.0 ± 0.0 g	395.0 ± 0.0 g	402.0 ± 0.0 g

*—statistically significant *p* value; G5^#^—data from the single surviving animal.

**Table 2:** Animal welfare mirrored by skin damage scores.

Groups	Skin Damage Score (Mean ± SD)					
	Day 7	Day 14	Day 21	Day 28	Day 35	Day 42
G1	0	0	0	0	0	0
G2	1.4 ± 0.8	1.0 ± 0.0	0.0 ± 0.0	0.0 ± 0.0	0.0 ± 0.0	0.0 ± 0.0
G3	1.1 ± 0.6	1.6 ± 0.9	0.8 ± 1.0	0.6 ± 0.7	0.3 ± 0.7	0.3 ± 0.7
G4	1.3 ± 0.6	0.7 ± 0.5	0.0 ± 0.0	0.0 ± 0.0	0.0 ± 0.0	0.0 ± 0.0
*p*-value	0.226	0.025 *	0.019 *	0.019 *	0.495	0.495
	**Day 30**	**Day 60**	**Day 90**	**Day 110**	**Day 130**	**Day 150**
G5^#^	4.0 ± 0.0	4.5 ± 0.0	5.0 ± 0.0	4.0 ± 0.0	3.5 ± 0.0	3.0 ± 0.0

*—statistically significant *p* value; G5^#^—data from the single surviving animal.

**Table 3:** Blood samples results.

Parameters	Unit	Normal Range	Group 1 (Control)	Group 2	Group 3	Group 4	Group 5^#^	Key Abnormalities	*p* (G1–G4)
Creatinine	mg/dL	0.2–1.2	0.81–0.93	0.69–0.89	0.7–0.93	0.64–0.88	0.5–0.81	Elevated in G3, G4, and G5	0.007
ASAT/TGO	g/L	37–205	80.3–92.4	87–125	97–165	82.8–108.4	61–83.7	Elevated liver markers in G3 and G5	0.002
ALAT/TGP	u/L	6–114	34.2–40.4	37.1–59	30.4–74.4	38.1–48.8	27–40.4	Elevated liver markers in G3 and G5	<0.001
Cholesterol	mg/dL	40–281	64–91	70–81	73–108	71–75	62–77	Normal	0.018
Glucose	mg/dL	56.1–197.2	153–185	60–211	0–908	148–222	144–163	Lowest glucose in G3 (significantly different from G1, G4; *p* < 0.001)	<0.001
Albumin	g/L	29–48	29.4–34.5	27.5–29.5	23.5–28.5	30.1–34.1	28.5–30.6	Low albumin in G3 and G5	<0.001
Total Protein	g/L	57–85	46.9–61.5	48–59	44.2–57.2	19.8–51.9	47–54.8	Low TP in G3 and G5	0.284
Urea	mg/dL	33.4–77.3	60.6–92.6	45–92	27.5–41.2	89.6–102.4	61–89.7	Elevated in G3, G4, and G5	<0.001
GGT	u/L	0–3	0	0	0	0	0	Normal	<0.001
Bile Acids	µmol/L	0–200	10–28	10–76	19–84	9–26	19–22	Elevated in G3	
RBC	million/mm^3^	7.1–9.6	8.02–8.68	6.5–8.7	6.7–8.5	8.44–8.96	6.71–8.55	Low RBC in G3 and G5	<0.001
Hemoglobin	g/dL	13.5–20.4	16.1–17.3	14–16.8	12.3–16.4	15.8–17.8	12.3–17.2	Low Hb in G3 and G5	<0.001
Hematocrit	%	36–50	44.3–47.6	37.5–48.4	36.5–46.3	44.5–49.4	36.5–47.3	Borderline low HCT in G3 and G5	0.003
Platelets	mm^3^	700,000–1,000,000	562,000–946,000	933,000–1,294,000	933,000–1,294,000	719,000–1,120,000	933,000–1,294,000	Elevated platelets in G3 and G5	0.126
WBC	mm^3^	5000–15,000	3930–5840	1520–6650	1520–6650	3780–5760	1520–6650	Low WBC in G3 and G5	0.381
Neutrophils	mm^3^	1200–7400	920–1850	640–2200	640–2200	1200–2280	640–2200	Low neutrophils in G3 and G5	0.650
Lymphocytes	mm^3^	3000–13,000	2790–3750	830–5510	830–5510	2400–3820	830–5510	Low lymphocytes in G3 and G5	0.784

Abbreviations: ASAT/TGO = aspartate aminotransferase; ALAT/TGP = alanine aminotransferase; GGT = gamma-glutamyl transferase; RBC = red blood cells; WBC = white blood cells. G5^#^—a single survival animal, not included in the statistical analysis between groups.

## Data Availability

Data from this study are available on request to the corresponding author.
